# Bcl-2 pathway inhibition in solid tumors: a review of clinical trials

**DOI:** 10.1007/s12094-022-03070-9

**Published:** 2023-01-13

**Authors:** Ioanna Ploumaki, Efthymios Triantafyllou, Ioannis-Alexios Koumprentziotis, Konstantinos Karampinos, Konstantinos Drougkas, Ioannis Karavolias, Ioannis Trontzas, Elias A. Kotteas

**Affiliations:** Oncology Unit, 3rd Department of Medicine, ‘Sotiria’ General Hospital, National and Kapodistrian University of Athens, Athens, Greece

**Keywords:** Bcl-2 inhibitors, Solid tumors, Chemotherapy, Venetoclax, Navitoclax, Oblimersen

## Abstract

Due to their key role in the pathogenesis of cancer through the regulation of apoptosis, the B-cell leukemia/lymphoma-2 (BCL-2) family proteins have been an attractive target for cancer therapy for the past decades. Throughout the years, many Bcl-2 family inhibitors have been developed, with Venetoclax being now successfully used in treating hematological malignancies. Although their effectiveness in the treatment of solid tumors is yet to be established, some preclinical evidence indicates their possible clinical application. This review aims to summarize current data from completed clinical trials that used Bcl-2 protein family inhibitors as monotherapy or in combination with other agents for the treatment of solid malignancies. We managed to include clinical trials of various phases which analyze the pharmacokinetics and pharmacodynamics of the drugs, as well as the effectiveness and adverse effects. Active and recruiting clinical trials are also briefly presented and future prospects and challenges are discussed.

## Introduction

Cancer is defined by unregulated cell division and growth. The main pathogenetic mechanisms leading to carcinogenesis include mutations in oncogenes or tumor-suppressor genes, epigenetic or chromosomal alterations and environmental stress, often resulting in the evasion of apoptosis [1, 2]. Apoptosis is a type of programmed cell death as a response to physiological processes, abnormal stimuli or cellular stress and, therefore, prevents a defective cell from evolving to cancer [3]. It occurs through two different pathways; the intrinsic, which is triggered by the release of apoptogenic factors in the mitochondria, and the extrinsic, which is activated upon ligation of specific death receptors at the plasma membrane [3, 4].

**Fig. 1:**
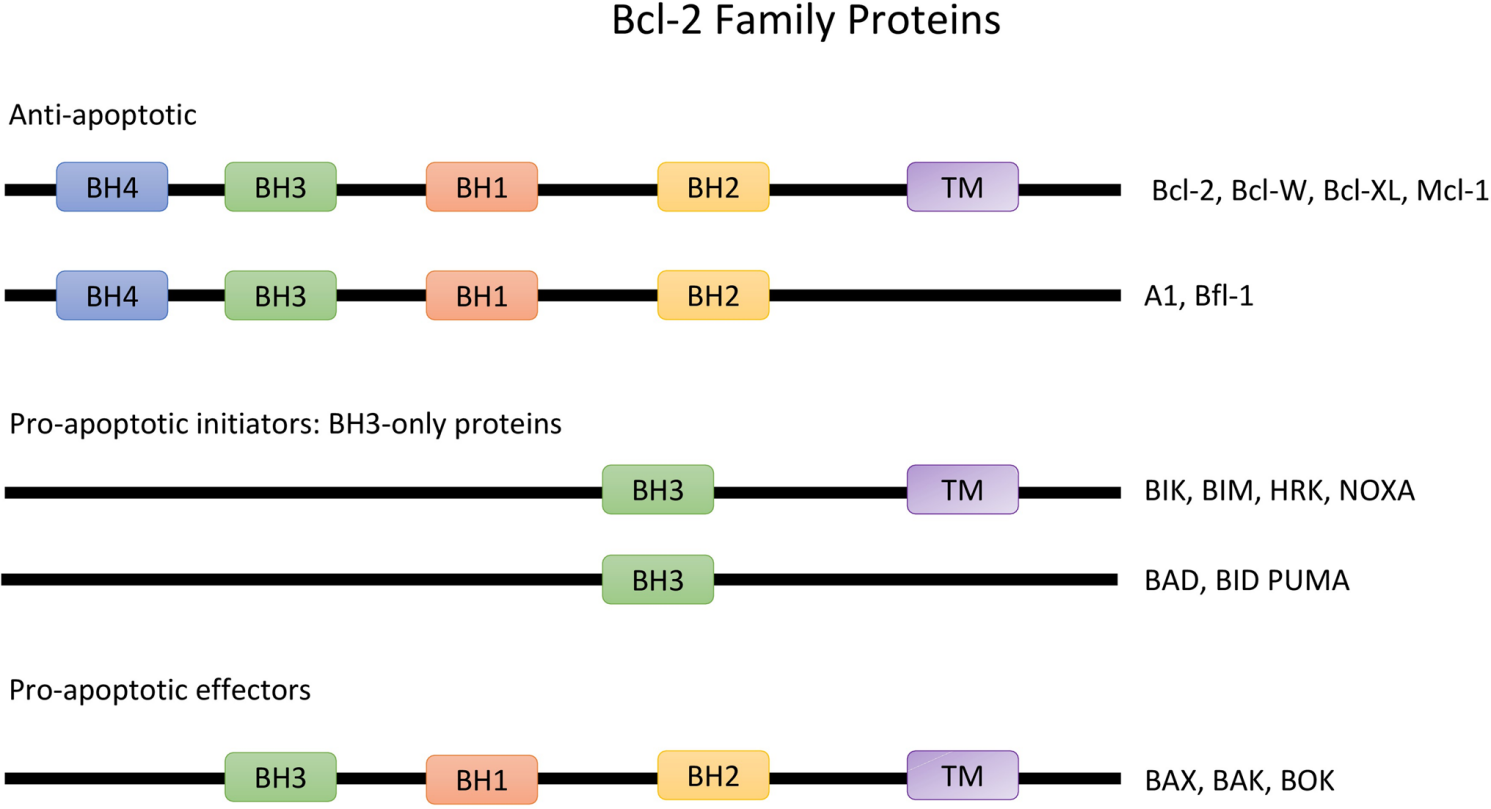
Illustration of Bcl-2 family of proteins which are classified based on morphology and Bcl-2 homology. The anti-apoptotic proteins possess four bcl-2 homology domains. The pro-apoptotic proteins contain three to four domains and the BH-3 only proteins possess only BH3 domains

**Fig. 2:**
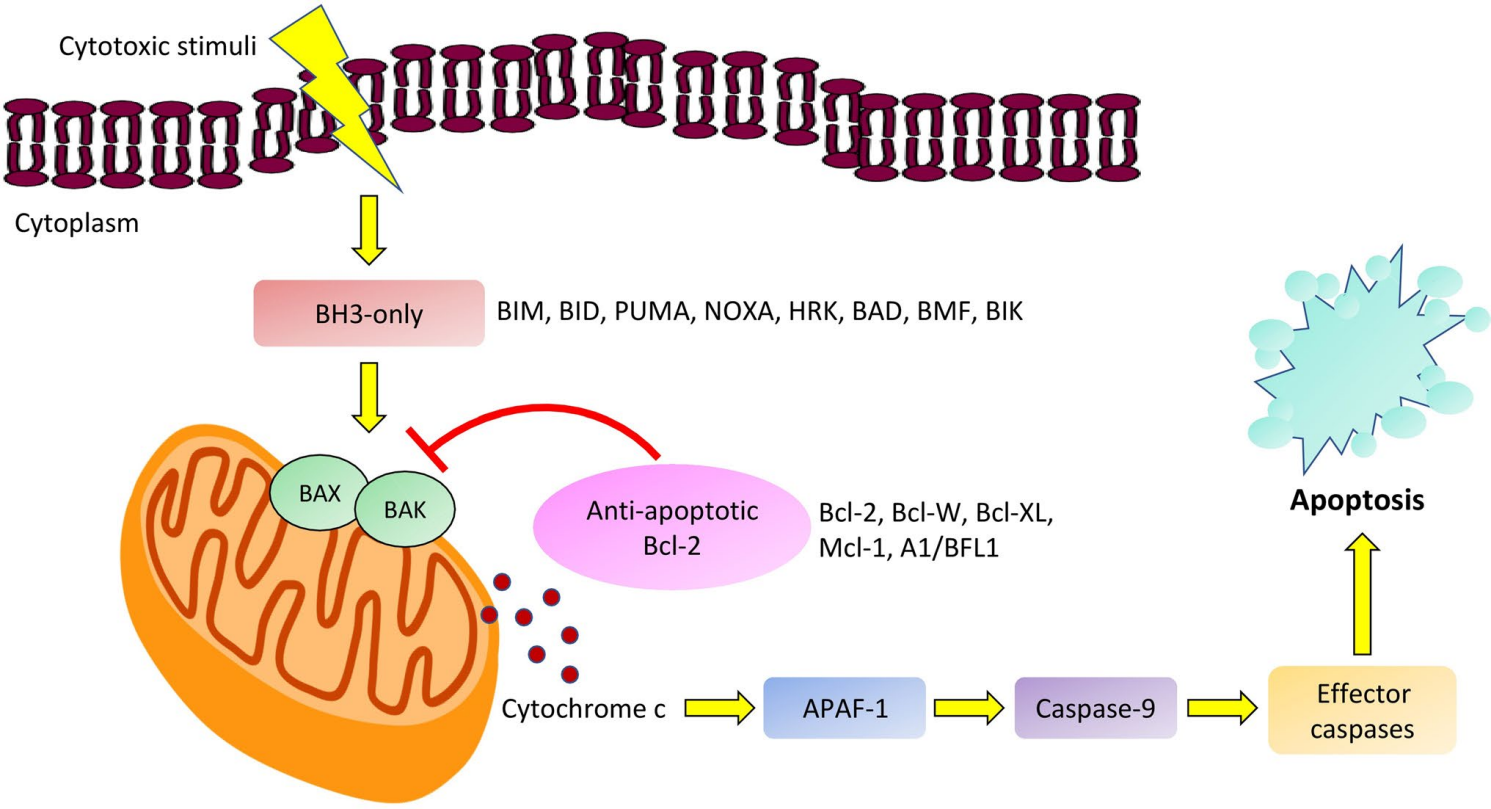
A model of the intrinsic pathway of apoptosis initiated by cytotoxic stimuli. The Bcl-2 family proteins exert their anti-apoptotic effect by inhibiting BAX, BAK and BH3-only proteins

**Fig. 3:**
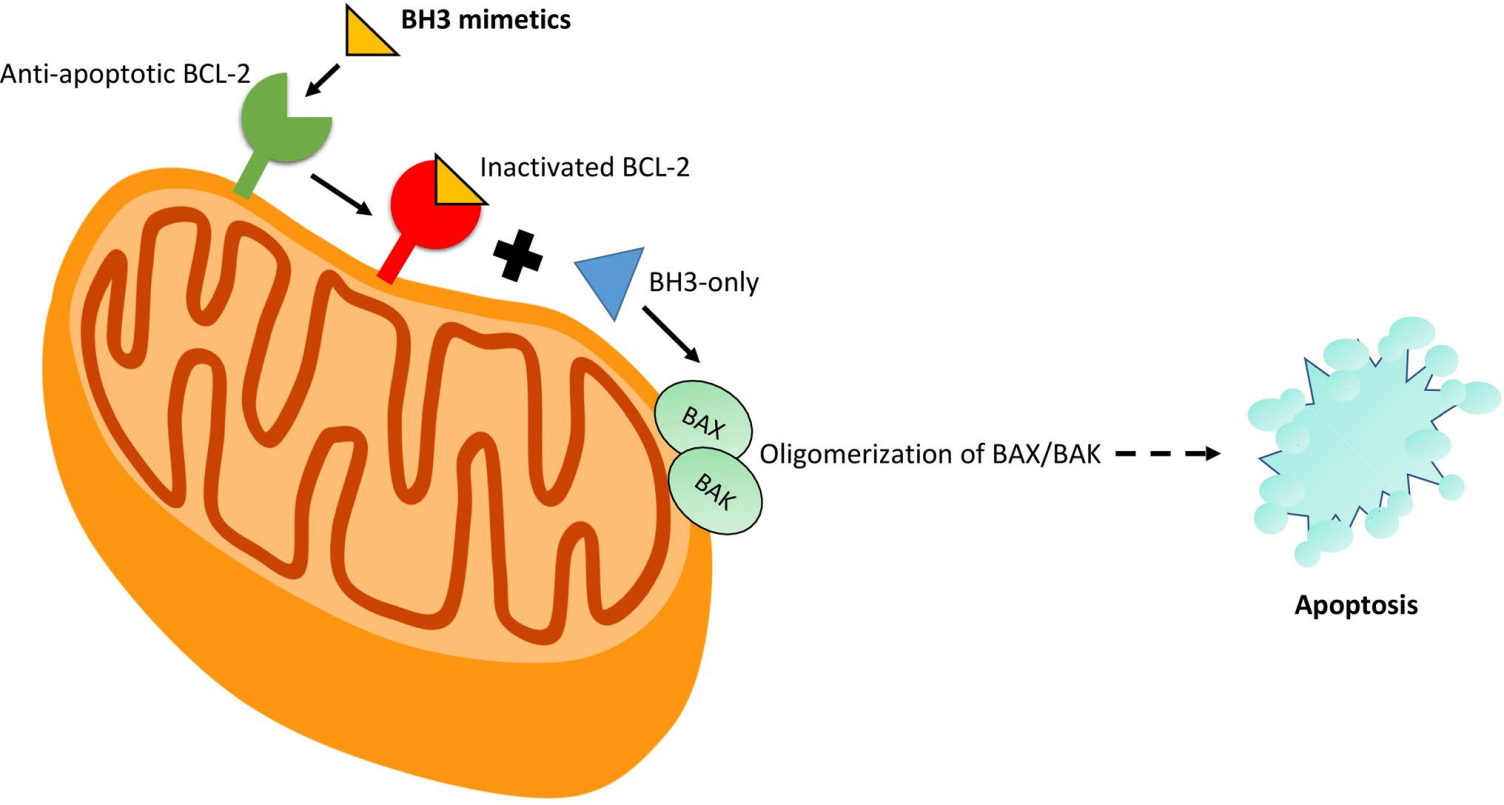
Brief overview of a BH3 mimetic route of action in order to initiate the apoptotic cascade through BAX and BAK (pro-apoptotic proteins) oligomerization

One of the main regulators of the intrinsic pathway is the B-cell leukemia/lymphoma-2 (Bcl-2), a family of regulatory proteins, which can be subclassified into different groups based on their morphology and Bcl-2 homology (BH) domain (Fig. 1). The BH3-only proteins activate BAX and BAK, which then form pores in the outer mitochondrial membrane, leading to the release of cytochrome c. This process initiates the caspase cascade pathway and eventually the apoptosis of the cell. The main action of the anti-apoptotic proteins is the inhibition of BAX, BAK and BH3-only proteins and therefore the apoptotic process [5, 6], (Fig. 2, Fig. 3).

It has been observed that in many types of cancer there is an up-regulation of anti-apoptotic proteins and a down-regulation of pro-apoptotic members of the Bcl-2 family [7]. The malignancies that were firstassociated with Bcl-2 overexpression were Chronic Lymphocytic Leukemia (CLL) and B-cell Lymphoma, hence the name Bcl-2 (B-cell leukemia/lymphoma-2 protein) [8]. Subsequently, researchers have been focusing on developing drugs that target the anti-apoptotic proteins, as an alternative approach to anticancer therapeutics. Many Bcl-2 protein family inhibitors have been developed over the past years, including venetoclax (ABT-199), navitoclax (ABT-263), obatoclax (GX15-070), oblimersen sodium (G3139), etc., and are mostly used in leukemia, lymphomas, and other hematological malignancies [9]. In particular, venetoclax was proved to be a major breakthrough in treating drug-resistant CLL, as it induces the intrinsic apoptotic cascade independently of TP53 expression [10].

Despite major breakthroughs in hematological neoplasms, the effectiveness of these drugs in solid tumors is still under investigation. Several clinical trials have been conducted regarding the role of different Bcl-2 protein family inhibitors in solid malignancies. In this narrative review we sought to provide an update on the available evidence from clinical trials of Bcl-2 inhibitors in solid malignancies and to briefly state the limitations, challenges and future goals of this anticancer approach.

## Clinical investigation of bcl-2 inhibitors in solid tumor treatment

### Oblimersen sodium (G3139)

Oblimersen sodium is an antisense oligonucleotide compound which targets the first 6 codons of the human Bcl-2 mRNA sequence, leading to degeneration of the Bcl-2 mRNA and decreased Bcl-2 protein production. It is the first oligonucleotide known to have an antisense effect on Bcl-2 protein translation [11]. Although it has not been approved by the Food and drug administration (FDA), many clinical trials suggest that oblimersen enhances the efficacy of cytotoxic chemotherapy especially for the treatment of CLL, multiple myeloma, malignant melanoma, and non-small cell lung cancer (NSCLC) [12]. Specifically for solid tumors, we found 16 completed clinical trials that evaluate the therapeutic potential of oblimersen in combination with other cytotoxic agents (Table 1).

**Table 1:** Completed clinical trials using Bcl-2 inhibitors in solid tumor treatment

Identifier/text reference*	Drug used	Trial design	Solid tumor/no of patients	Intervention/treatment	Results	Toxicity
NCT00004870/ [16]	Oblimersen sodium (G3139)	Multicenter, dose-escalation Phase I/II study	Colorectal cancer *n* = 73	Continuous IV administration of G3139 on days 1–7 and irinotecan IV over 90 min on day 6, on a 21-Day cycle	1 PR was observed 10 patients had SD for 2,5–10 months	Neutropenia, diarrhea, nausea, vomiting, fever and fatigue
NCT00017251	Oblimersen sodium (G3139)	Open-Label Phase I study	Extensive stage SCLC *n* = 12	Patients receive G3139 IV continuously on days 1–8, carboplatin IV over 30 min on day 6, and etoposide IV over 1 h on days 6–8 on a 21-Day cycle	PR was observed in 12 patients 2 patients had SD	Grade 4 neutropenia
NCT00039481	Oblimersen sodium (G3139)	Open-label phase I study	Unspecified childhood solid tumor *n* = 15	Part A: Patients receive oblimersen IV continuously on days 1–7. Also they receive dexrazoxane IV followed by doxorubicin IV over 15 min followed by cyclophosphamide IV over 1 h on days 5 and 6. Additionally, Filgrastim (G-CSF) subcutaneously once daily beginning on day 8 and continuing until blood counts recover Part B: oblimersen at the MTD and escalating doses of dexrazoxane, doxorubicin, and cyclophosphamide on the same schedule as in part A	NA	NA
NCT00085228/ [21]	Oblimersen Sodium (G3139)	Randomized, multicenter Phase II study	Prostate cancer *n* = 116	Arm I: Docetaxel IV over 1 h on day 5 and oblimersen IV continuously on days 1–7 on 21-Day cycle Arm II: Docetaxel IV over 1 h on day 1	PSA response of 37% in Arm I and 46% in Arm II PR was at 24% in Arm I and 18% in Arm II	Grade 3 or 4 fatigue, myositis and thrombocytopenia
NCT00005032	Oblimersen sodium (G3139)	Open-label phase I/II study	Recurrent SCLC *n* = 12	G3139 (3 mg/kg/day continuous IV infusion over 7 days every 21 days), Paclitaxel (150 mg/m2, 3 h IV infusion on Day 6 of every 21 day cycle)	2 patients had SD There was no objective response observed	Grade 3 leukopenia, neutropenia, thrombocytopenia, anemia and Grade 4 respiratory toxicity
NCT00042978	Oblimersen sodium (G3139)	Parallel assignment, randomized Open-Label Phase II study	Extensive Stage and Recurrent SCLC *n* = 55	Arm I: G3139 (IV) continuously on days 1–8, carboplatin IV over 30 min on day 6, and etoposide IV over 60 min on days 6–8 on 21-Day cycle Arm II: Carboplatin IV over 30 min on day 1 and etoposide IV over 60 min on days 1–3	RR was 61% in Arm I and 60% in Arm II Lower OS in Arm I than in Arm II	Grade 3 to 4 hematologic toxicity
NCT00017251	Oblimersen sodium (G3139)	Open-label phase I study	Extensive Stage and Recurrent SCLC *n* = 16	G3139 IV continuously on days 1–8, carboplatin IV over 30 min on day 6, and etoposide IV over 1 h on days 6–8 on a 21-Day cycle	PR in 12 patients SD in 2 patients Median time to progression = 5.9 months	Grade 4 neutropenia, Grade 3 thrombocytopenia
NCT00055822	Oblimersen sodium (G3139)	Open-label, dose-escalation phase I study	Colorectal cancer *n* = 16	Oblimersen IV continuously on days 1–5 and 15–19; leucovorin calcium IV over 2 h and fluorouracil IV over 22 h on days 6, 7, 20, and 21; and oxaliplatin IV over 2 h on days 6 and 20	NA	NA
NCT00059813	Oblimersen sodium (G3139)	Open-label phase II study	Metastatic renal cancer *n* = 41	Oblimersen, 7 mg/kg/day IV 7 days of every 14 day cycle, plus alpha-IFN, 5 million units/m(2) subcutaneously, days 4 and 6 of the first oblimersen infusion, then thrice weekly	PR in 1 patient lasting 2.5 months	Grade 3–4 fatigue, fever, myelosuppression, hepatic enzyme and metabolic abnormalities
NCT00636545	Oblimersen sodium (G3139)	Open-label phase I study	Solid tumors* n* = 25	In Part 1, Cohort 1 receives a once weekly short IV infusion for 3 weeks at a starting dose of 300 mg and increasing in increments of 100 mg to the MTD; Cohort 2 receives a corticosteroid prior to the once-weekly short IV infusion for 3 weeks In Part 2, pts receive a twice weekly short IV infusion at the MTD determined in Cohort 1	At 900 mg a patient had syncope At 1000 mg, all 6 patients had fever, chills, and moderate decreases in blood pressure	Syncope, fever, chills, decrease in blood pressure
NCT00047229	Oblimersen sodium (G3139)	Open-label phase II study	Advanced HCC *n* = 27	G3139 was escalated from 5 to 7 mg/kg for 7 days continuous IV infusion on Day 1–8 and doxorubicin was escalated from 45 to 60 mg/m2 IV bolus on Day5, every 28 days (in three cohorts)	SD in 6 patients Median time to progression was 1.8 months 18 of 19 patients have died with median survival of 5.4 months	Grade 4 neutropenia, lymphopenia, thrombocytopenia, transaminitis, and grade 1–2 fever
NCT00054548	Oblimersen sodium (G3139)	Open-label phase I study	Unspecified adult solid tumor *n* = 55	Oblimersen IV continuously on days 1–7 and paclitaxel IV over 3 h and carboplatin IV over 30 min on day 4 on a 21-Day cycle Cohorts of 3–6 patients receive escalating doses of oblimersen until MTD	NA	NA
NCT00003103	Oblimersen sodium (G3139)	Phase I/IIA study	Androgen-independent prostate cancer or other advanced solid tumor malignancies *n* = 57	Phase I: G3139 IV on days 1–5 and docetaxel IV on day 5 on 21-Day-cycle Phase II: G3139 IV continuously over 21 days at one dose level below the MTD in combination with weekly docetaxel	NA	NA
NCT00079131	Oblimersen sodium (G3139)	Open-label phase II study	Merkel cell carcinoma *n* = 37	Patients receive oblimersen IV continuously (7 mg/kg/d) on days 1–14 on a 21-Day cycle	SD in 3 patients PD in 9 patients	Grade 4 lymphopenia, hyperkalemia, grade 3 renal failure, cytopenia, AST and ALT elevation, hypophosphatemia and pain
NCT00409383	Oblimersen sodium (G3139)	Open-label phase I study	Melanoma *n* = 28	Arm I: 56-day cycles of oblimersen (7 mg/kg/day continuous IV infusion on day 22–28) temozolomide (75 mg/m(2), days 1–42), and nab-paclitaxel (175 mg/m(2) on day 7 and 28) Arm II: 56-day cycles of oblimersen (7 mg/kg/day continuous IV infusion on day 22–28), temozolomide (75 mg/m(2), days 1–42), and nab-paclitaxel (260 mg/m(2) on days 7 and 28) Arm III: 56-day cycles of oblimersen 900 mg fixed dose, twice weekly in weeks 1–2, 4–5. temozolomide (75 mg/m(2), days 1–42), and nab-paclitaxel (175 mg/m(2) on day 7 and 28)	Objective RR was 40.6% 11 patients had SD, for a disease control rate of 75%	Grade 4 neutropenia and thrombocytopenia, grade 3 renal insufficiency, hyponatremia, elevated creatinine, allergic reaction, and neuropathy
NCT00016263/ [18]	Oblimersen sodium (G3139)	Phase III study	Advanced malignant melanoma *n* = 771	Arm I: dacarbazine (1,000 mg/m2) preceded by a 5-day continuous intravenous infusion of oblimersen sodium (7 mg/kg/d) every 3 weeks for up to eight cycles Arm II: dacarbazine (1000 mg/m2) alone	Arm I vs Arm II Survival at 24-month minimum follow-up (median, 9.0 vs. 7.8 months, *P* = .077) PFS (median, 2.6 vs. 1.6 months, *P* < .001) OR (13.5% vs. 7.5%, *P* = .007) CR (2.8% vs. 0.8%), and durable response (7.3% vs. 3.6%; *P* = .03) Oblimersen significantly increased survival in patients whose baseline serum LDH was not elevated (median OS, 11.4 vs. 9.7 months; *P* = .02)	Grade 3–4 Neutropenia and thrombocytopenia
NCT00521144/ [18]	Obatoclax mesylate (GX15-070)	Open-label Phase II Study	Relapsed SCLC *n* = 9	Patients receive obatoclax mesylate IV over 3 h on day 1 OR days 1 and 3 and topotecan hydrochloride IV over 30 min on days 1–5 of a 21-day cycle	There were no PR’s or CR’s 5 had SD 4 patients developed PD	Grade 3 or 4 thrombocytopenia (22%), anemia (11%), neutropenia (11%) and ataxia (11%)
NCT00405951/ [25]	Obatoclax mesylate (GX15-070)	Open-label phase I/II study	Relapsed NSCLC *n* = 18 (phase I) *n* = 32 (phase II)	Phase I: Docetaxel as a 1-h infusion on day 1 and obatoclax as a 24-h infusion on days 1 and 2—every 3 weeks for up to eight cycles Docetaxel 75 mg/m2 and obatoclax 60 mg (median 2 cycles)	SD was the best response in 11 of 18 patients in Phase I 3 patients (11%) had PR in phase 2 2 demonstrated SD lasting 12 weeks or more Median duration of response was 4.8 months Median PFS was 1.4 months	2 DLT’s occurred during Phase I: one febrile neutropenia each at dose levels 3 and 4 Grade 3 or 4 neutropenia (31%), febrile neutropenia (16%), and dyspnea (19%) in phase II
NCT00682981/ [27]	Obatoclax mesylate (GX15-070)	Randomized phase II study	Extensive-stage SCLC *n* = 155	Arm I/ CbEOb: obatoclax (30 mg infused over 3 h on days 1–3), followed by 132 carboplatin on day 1 and etoposide on days 1–3 Arm II/ CbE: carboplatin AUC 5 was administered on day 1 followed by etoposide 100 mg/m^2^ 130 on days 1–3	ORR was 62% with CbEOb versus 53% with CbE (1-sided *p* = 0.143) Median PFS and OS were 5.8 months (95% CI 5.3–6.5) and 10.5 months (8.9–13.8) with CbEOb and 5.2 months (95% CI 4.1–5.7) and 9.8 months (7.2–11.2) with CbE	Grade 3/4 hematologic adverse events, similar in frequency between treatment arms
NCT00682981	Obatoclax mesylate (GX15-070)	Open-label, phase I	Extensive-stage SCL *n* = 25	Arm I: 3-h infusion of obatoclax at a dose of 15, 30 or 45 mg (on days 1–3), followed by an IV infusion of carboplatin (AUC 5; day 1 only) and of etoposide (100 mg m − 2; days 1, 2, and 3) on a 21-day cycle Arm II: 24-h infusion of obatoclax at a dose of 30, 45 or 60 mg (on days 1–3), followed by an IV infusion of carboplatin (AUC 5; day 1 only) and of etoposide (100 mg m − 2; days 1, 2, and 3) on a 21-day cycle	MTD was established with the 3-h infusion at 30 mg per day and was not reached with the 24-h infusion The ORR was 68% (81 vs. 44% in the 3-h and the 24-h infusion cohorts, respectively) The median OS was numerically higher in patients who received obatoclax by 3-h infusion (379 vs. 283 days)	Neutropenia (96%), thrombocytopenia (76%), anemia (72%), fatigue (68%), and nausea. Compared with the 24-h cohorts, the 3-h cohorts had higher incidence of CNS adverse events
NCT00521144	Obatoclax mesylate (GX15-070)	Open-label phase I study	Solid tumors *n* = 14	Patients received obatoclax mesylate and topotecan on a 3-week cycle in a pre-defined, standard 3 + 3 dose escalation scheme. The starting dose for obatoclax mesylate was 14 mg/m^2^ by 3-h IV infusion. Topotecan 1.25 mg/m^2^ was given concurrently as an IV infusion on days 1–5 of each cycle	Two patients with SCLC achieved PR and 4 patients had SD Median time to progression was 12 weeks Two of five patients experienced dose-limiting grade 3 neurologic toxicity and febrile neutropenia at a dose of 20 mg/m^2^	Grade 3 somnolence, speech impairment, ataxia, mood disturbance and febrile neutropenia
NCT0252077/ [31]	Navitoclax (ABT-263)	Open label, single-arm, phase IB	EGFR-positive NSCL *n* = 27	Patients receive navitoclax PO QD on days 1–28 and osimertinib PO QD on days 4–28 (days 1–28 during dose-expansion). Cycles repeat every 28 days in the absence of disease progression or unacceptable toxicity	RP2D: osimertinib 80 mg, navitoclax 150 mg OR 100% Median-PFS: 16.8 months No DLTs were seen in dose-escalation cohort	Grade 2–3 fatigue, leukopenia, thrombocytopenia and diarrhea
NCT00878449	Navitoclax (ABT-263)	Open label, phase I study	Solid tumors *n* = 12	150 mg of ABT-263 is taken daily for 3 out of 21 days. This is a dose escalation study; therefore, the dose of ABT-263 will change throughout the study. etoposide = 100 mg/m^2^ Days 1–3 of each Cycle; Max duration 6 cycles. cisplatin = 75 mg/m^2^ Day 1 of each Cycle; Max duration 6 cycles	NA	NA
NCT02591095/ [30]	Navitoclax (ABT-263)	Prospective multicenter single-arm Phase II study	Platinum-resistant or Refractory Ovarian cancer *n* = 47	Navitoclax was administered as monotherapy at the daily dose of 150 mg during a lead-in period (7–14 days) and then increased to 250 mg daily in the absence of dose-limiting thrombocytopenia (< G3)	3-month PFS = 22.7% and median PFS = 1.64 months 1 PR 15 SD	Grade 3/4 thrombocytopenia
NCT00891605	Navitoclax (ABT-263)	Open Label, Phase I study	Solid tumors *n* = 19	Arm A: 150 mg navitoclax orally, 30 min after the completion of breakfast, for 3 or 5 consecutive days in a 21-day cycle. Paclitaxel (175 mg/m2) was administered via IV infusion over 3 h, followed by IV infusion of carboplatin (AUC 4–6) over 1 h on Day 1 of each cycle Arm B: 150 mg navitoclax orally, 30 min after the completion of breakfast, for 3 or 5 consecutive days in a 21-day cycle with Paclitaxel (135 mg/m2 or 175 mg/m2) via IV infusion over 3 h on Day 1 of each cycle	No PK interaction between navitoclax and paclitaxel/carboplatin 1 PR	Alopecia, Grade 3/4 thrombocytopenia, neutropenia
NCT00887757	Navitoclax (ABT-263)	Open label, phase I study	Solid tumors *n* = 46	Arm A: On a 21-day dosing schedule navitoclax was administered orally on days 1–3 and 8–10; and gemcitabine 1,000 mg/m(2) on days 1 and 8 Arm B: On a 28-day dosing schedule navitoclax was administrated orally on days 1–3, 8–10, and 15–17; and gemcitabine 1,000 mg/m(2) on days 1, 8 and 15	MTD of navitoclax was 325 mg No objective responses. SD, reported at the end of cycle 2, was the best response in 54% of evaluable patients (*n* = 39) No PK interaction between navitoclax and gemcitabine	Grade 4 thrombocytopenia, neutropenia and grade 3 AST elevation
NCT00445198/ [23]	Navitoclax (ABT-263)	Open label phase I study	SCLC *n* = 86	Arm A: intermittent dosing, 14 days on drug, 7 days off Arm B: continuous dosing, received a 1-week lead-in dose of 150 mg followed by continuous daily administration	1 patient with SCLC had a confirmed PR lasting longer than 2 years 8 patients with SCLC or carcinoid had SD	Thrombocytopenia (dose-depended), diarrhea, nausea, vomiting and fatigue
NCT00888108	Navitoclax (ABT-263)	Open label, phase I study	Solid tumors *n* = 41	Arm A: navitoclax (150 or 200 mg) was administered p.o. once daily as a liquid formulation via syringe on days 1–5 or 1–3 every 21 days with docetaxel 75 mg/m2 administered via IV infusion over 1 h on day 1 Arm B: navitoclax (150 or 200 mg) was administered PO QD on days 1–3, 8–10 and 15–17 every 28 days with iv. docetaxel 30 mg/m2 on days 1, 8 and 15	Navitoclax 150-mg days 1–5 every 21 days with docetaxel 75 mg/m2 day 1 was the MTD and optimal schedule 4 confirmed PR’s 10 patients experienced DLT’s	Thrombocytopenia, fatigue, nausea, neutropenia
NCT01009073	Navitoclax (ABT-263)	Non-Randomized, Parallel assignment, Open Label Phase I study	Solid tumors *n* = 11	Dose escalation study included an arm evaluating navitoclax combined with erlotinib, which included a dose escalation cohort and a planned safety expansion cohort. Patients with documented cancers for whom erlotinib therapy was appropriate, received erlotinib 150 mg orally once daily plus navitoclax 150 mg orally once daily, with navitoclax dose escalation via a continuous reassessment method model	No objective responses were observed, disease control rate was 27% No PK interaction between navitoclax and erlotinib DLTs in 4 patients, most commonly diarrhea	Diarrhea, nausea, vomiting, and decreased appetite
NCT01121133	Navitoclax (ABT-263)	Non-randomized, open label phase I study	Lymphoma, CLL, solid tumors *n* = 12	2-period study. 7-day washout period separated the two treatment periods On Study Day 1 and Day 8- > 250 mg oral dose of navitoclax. Rifampin 600 mg- > once daily (QD) on Study Day 4 -Day 10	Co-administration navitoclax with rifampin moderately decreased navitoclax AUC	Diarrhea, nausea, vomiting and thrombocytopenia
NCT01021358	Navitoclax (ABT-263)	Open label, phase I study	Lymphoma, CLL, solid tumors *n* = 12	Single doses of navitoclax at 60 mg orally on days 1 and 8. Ketoconazole at 400 mg once daily from days 7 through 10	Navitoclax exposure with co-administration of ketoconazole did not increase above that observed with navitoclax monotherapy	No AE related to navitoclax exposure
NCT01009073	Navitoclax (ABT-263)	Non-randomized, parallel assignment, open label phase I study	Advanced solid tumors *n* = 31	Arm A: dose escalation study with navitoclax (starting dose 150 mg/day) in combination with irinotecan once-every-3-week regimen (Q3W 180, 250, or 350 mg/m(2)) Arm B:: dose escalation study with navitoclax (starting dose 150 mg/day) in combination with irinotecan once-weekly regimen (QW 75 or 100 mg/m(2))	In the QW group, MTD and RP2D for navitoclax were 150 mg with irinotecan 75 mg/m(2) administration PR in 2 patients (1 from each group) No PK interactions between navitoclax and irinotecan	Grade 3/4 diarrhea
NCT00445198/ [29]	Navitoclax (ABT-263)	Open label phase II study	SCLC *n* = 39	Daily administration of Navitoclax in a dose of 325 mg, following an initial lead-in of 150 mg daily for 7 days	PR was observed in 1 patient and SD in 9 patients Association between plasma pro-gastrin-releasing peptide and tumor Bcl-2 copy number (R = 0.93)	Grade 3/4 thrombocytopenia, neutropenia, AST and ALT elevations, diarrhea, nausea and fatigue
NCT03080311/ [34]	Palcitoclax (APG-1252)	Open label, single group assignment phase I study	SCLC, others *n* = 50	Arm 1: 30-min IV infusion twice weekly of APG-1252, 28-day cycle, start dose is 10 mg, dose escalation to 240 mg twice weekly Arm 2: 30-min IV infusion twice weekly of APG-1252, 28-day cycle, start dose is 240 mg	3 patients with PR 7 patients achieved SD 26 patients had PD DLT: 320–400 mg	Grade 4 thrombocytopenia, Grade 1 or Grade 2 AST and ALT elevation
NCT01633541/ [43]	AT-101 (R-(-)-gossypol acetic acid)	Open-label phase II study	Advanced laryngeal cancer *n* = 54	Arm I: Day 1; docetaxel 75 mg/m2 and cisplatin 100 mg/m2. Days 1–3; AT-101 40 mg orally twice daily On Day 23 (± 3 days) Arm II: Day 1; docetaxel 75 mg/m2 and cisplatin 100 mg/m2	Organ preservation rate was 62% The 2 year laryngectomy-free survival is 54% (95% CI 38–68) & 2 year OS is 81% (95% CI 64–90) in both Arms	Grade 3–4 nausea, neutropenia and infection
NCT00666666	AT-101 (R-(-)-gossypol acetic acid)	Open-label phase II study	Adenocarcinoma of the prostate *n* = 55	AT101 will be administered orally 20 mg/day for 21 days of a 28 day cycle Hormone therapy with at least one LHRH agent (Leuprolide Acetate or Goserelin) for 6 weeks plus bicalutamide	An undetectable PSA level was achieved in 31% of the patients Treatment was discontinued in 35% (19/55) of patients due to adverse events	Grade 3 Sensory neuropathy, ileus, small intestine obstruction and syncope
NCT00571675/ [42]	AT-101 (R-(-)-gossypol acetic acid)	Randomized, double-blind, placebo-controlled, multicenter, phase II Study	Hormone refractory prostate cancer *n* = 220	Patients received docetaxel (75 mg/m2 day 1) and prednisone 5 mg orally twice daily every 21 days with either AT-101 (40 mg) or placebo twice daily orally on days 1–3	Median OS was 18.1 months with AT-101 versus 17.9 months with placebo In metastatic castration-resistant prostate cancer outcomes favored AT-101 scheme (median OS 19 versus 14 months)	Grade 3/4 toxic effects for AT-101 versus placebo were cardiac events (5% versus 2%), lymphopenia (23% versus 16%), neutropenia (47% versus 40%), ileus (2% versus 0%) and pulmonary embolism (6% versus 2%)
NCT00286806	AT-101 (R-(-)-gossypol acetic acid)	Open-Label, Multicenter, Phase I/II Study	Hormone refractory prostate cancer *n* = 27	Escalating doses of AT-101 on a continuous daily basis until the MTD Starting dose was 30 mg/day	2 patients had a confirmed > or = 50% post-therapy PSA decline No objective responses were observed	Grade 3 small intestinal obstruction, and any grade of diarrhea, fatigue, nausea, anorexia
NCT00397293	AT-101 (R-(-)-gossypol acetic acid)	Open label, multicenter phase I/II study	SCLC *n* = 36	Oral AT-101 with intravenous topotecan Phase II: 40 mg AT-101 days 1–5 with topotecan 1.25 mg/m(2) days 1–5 on a 21-day cycle 2 cohorts of patients, sensitive relapsed and refractory	Sensitive-relapsed cohort (*n* = 18), 0 CR, 3 PR, 10 SD, and 4 PD In the refractory cohort, there were 0 CR/PR, 5 SD, and 5 PD Median time to progression in the sensitive-relapsed cohort was 17.4 weeks and 11.7 weeks in the refractory cohort	Grade 3 elevation in lactate dehydrogenase, and grade 3 elevation in gamma-glutamyl transferase
NCT00544960/ [41]	AT-101 (R-(-)-gossypol acetic acid)	Randomized, 2-Arm, double-blind phase II study	NSCLC *n* = 106	Arm I: AT-101 30 mg on days 1, 2, and 3 of each 21-Day cycle, docetaxel, 75 mg/m^2^ on day 1 of each 21 day cycle Arm II: placebo 3 tabs on days 1, 2, and 3 of each 21 day cycle, docetaxel, 75 mg/m^2^ on day 1 of each 21 day cycle	PFS 7.5 weeks for docetaxel plus AT-101 and 7.1 weeks for docetaxel plus placebo(HR, 1.04; *p* = 0.57) Median OS was 7.8 months for docetaxel plus AT-101 versus 5.9 months for docetaxel plus placebo (HR 0.82; *p* = 0.21)	Fatigue, anemia, and dyspnea
NCT00773955	AT-101 (R-(-)-gossypol acetic acid)	Open-label phase II study	Extensive Stage and Recurrent SCLC *n* = 15	Patients receive oral AT-101 once daily on days 1–21. Courses repeat every 28 days	0 responses to the treatment 3 patients had SD after 2 cycles	Grade 3 anorexia, fatigue, and nausea/vomiting
NCT00544596	AT-101 (R-(-)-gossypol acetic acid)	Open-label phase I study	Extensive stage SCLC, unspecified adult solid tumor *n* = 27	Oral AT-101 twice daily on days 1–3, cisplatin IV over 60 min on day 1*, and etoposide IV over 30 min on days 1*-3. Treatment repeats every 21 days	Preliminary activity was observed with PRs in patients with extensive stage SCLC, high-grade neuroendocrine tumor, esophageal cancer and NSCLC	Grade 3/4 diarrhea, increased AST, neutropenia, hypophosphatemia, hyponatremia, myocardial infarction and pulmonary embolism
NCT00286793	AT-101 (R-(-)-gossypol acetic acid)	Open-label, Multicenter, Phase I/II Study	Hormone refractory prostate cancer *n* = 76	Docetaxel (75 mg/m2 q 3 weeks) in combination with Prednisone (5 mg/BID on days 1–21), and AT-101 at 40 mg/BID on days 1–3 of each cycle	Ten patients had a decline in PSA ≥ 50% 12 patients had a decline in PSA ≥ 30%	Grade 4 Neutropenia
NCT01977209	AT-101 (R-(-)-gossypol acetic acid)	Randomized, Double Blind, Placebo-controlled Multiple-center Phase III Study	Advanced NSCLC With APE1 High Expression *n* = 106	Arm I: Patients received 40 mg b.i.d. × 3 days of AT-101 with 75 mg/m of docetaxel on day 1 every 21 days Arm II: Patients received placebo and 75 mg/m of docetaxel on day 1 every 21 days	There was no significant improvement in RR for AT-101 compared with placebo There was no increase for grade 3 and 4 toxicity for AT-101 compared with placebo	Grade 3/4/5 fatigue, dyspnea, anemia, neutropenia and leukopenia
NCT00390403	AT-101 (R-(-)-gossypol acetic acid)	Open-Label Phase I Study	Brain and CNS tumors *n* = 50	Arm I: Patients receive oral AT-101 and undergo radiotherapy once daily 5 days a week for up to 6 weeks. Plus oral temozolomide once daily for up to 6 weeks Arm II: Patients receive oral temozolomide on days 1–5 and oral AT-101 once daily on days 1–21 (28-Day cycle)	NA	NA
NCT00848016	AT-101 (R-(-)-gossypol acetic acid)	Open-label phase II study	Advanced Adrenocortical Carcinoma *n* = 29	20 mg oral AT-101 once daily on days 1–21. Treatment repeats every 28 days in the absence of disease progression or unacceptable toxicity	No PR was observed	Grade 4 toxicity cardiac troponin elevations and hypokalemia
NCT00540722	AT-101 (R-(-)-gossypol acetic acid)	Open-label phase II study	Progressive or Recurrent Glioblastoma Multiforme *n* = 56	Patients receive oral AT-101 once daily on days 1–21. 28-Day cycle in the absence of PD or unacceptable toxicity	CR 0.0% PR 1.8% SD 26.8% PD 62.5%	Elevated troponin, ileus, hypophosphatemia, fatigue and seizures
NCT01285635	AT-101 (R-(-)-gossypol acetic acid)	Open-label phase II study	Squamous Cell Carcinoma of the Head and Neck *n* = 35	Arm I: Docetaxel 75 mg/m2 on Cycle Day 1 Arm II: Pulse Dose: AT-101 dose of 40 mg b.i.d. on days 1–3 and docetaxel 75 mg/m2 on Cycle Day 1 Arm III: Metronomic Dose: AT-101, 20 mg daily, days 1–14 and docetaxel 75 mg/m2 on Cycle Day 1	ORR was 11% with a clinical benefit rate of 74% Median PFS was 4.3 months (range: 0.7–13.7) OS was 5.5 months (range: 0.4–24) No significant differences were noted between dosing strategies	Grade 3–4 lymphopenia, anemia. No differences in treatment tolerability or toxicities were grossly apparent
NCT03584009/ [48]	Venetoclax (ABT-199)	Multicenter, open-label, randomized Phase II Study	ER + /Human epidermal growth factor receptor negative locally advanced or metastatic breast cancer *n* = 103	Arm I: Participants were administered Venetoclax 800 mg orally once daily and Fulvestrant 500 mg IM on Day 1 and 15 of Cycle 1 and Day 1 of subsequent cycles (Cycle length = 28 days) Arm II: Participants were administered Fulvestrant 500 mg only IM on Day 1 and 15 of Cycle 1 and Day 1 of subsequent cycles (Cycle length = 28 days)	Clinical benefit 11.8% in Arm I and 13.7% in Arm II (*P*-Value = 0.7286) PFS was 2.69 months in Arm I and 1.94 months in Arm II (*P*-Value = 0.785) OR was 3.9% in Arm I and 5.9% in Arm II (*P*-Value = 0.5978) Serious AE was 8% in Arm I and 1.96% in Arm II	Pyrexia, Lower respiratory tract infections, urosepsis, decreased ejection fraction, flank pain and pleural effusion
NCT03000257	Venetoclax (ABT-199)	Open-label, phase I	Advanced solid tumors *n* = 182	Part I: Venetoclax will be taken once daily beginning 7 days prior to cycle 1 and continuing daily for a 28 day cycle and ABBV-181 Part II: ABBV-181 will be administered at escalating dose levels in 28-day dosing cycles (2 doses per cycle) Part III: Rovalpituzumab Tesirine will be given once every six weeks times two doses and ABBV-181 will be administered every 3 weeks	NA	NA

*IV* intravenous, *mg* milligram, *OR* overall response, *PR* partial response, *SD* stable disease, *RR* response rate, *OS* overall survival, *PFS* progression- free survival, *NA* not available, *SCLC* small-cell lung cancer, *G-CSF* granulocyte colony stimulating factor, *MTD* maximum tolerated dose, *PSA* prostate specific antigen, *IFN* interferon, *HCC* hepatocellular carcinoma, *LDH* lactate dehydrogenase, *PD* progressive disease, *CR* complete response, *ORR* overall response rate, *CNS* central nervous system, *CI* confidence interval, *DLT* dose-limiting toxicity, *RP2D* recommended phase 2 dose, *PK* pharmacokinetic, *PO* per os, *QD* quaque die(once daily), *AUC* area under the curve, *CLL* chronic lymphocytic leukemia, *AE* adverse event, *LHRH* Luteinizing hormone-releasing hormone, *HR* hazard ratio, *AST* aspartate aminotransferase, *ALT* alanine transaminase, *APE1* apurinic/apyrimidinic endodeoxyribonuclease 1, *ER* estrogen receptor, *IM* intramuscular

*References are used for clinical trials which are cited and commented in the text

In a phase I dose-escalation, multicenter study in 2000 oblimersen sodium was combined with irinotecan in patients with metastatic colorectal carcinoma to assess the pharmacokinetic behavior and the Bcl-2 protein inhibition in peripheral blood mononuclear cells (PBMCs), since preclinical studies have detected Bcl-2 overexpression in colorectal carcinoma specimens compared to normal colonic epithelium [13–15]. The co-administration of oblimersen and irinotecan was found to be well tolerated and moderately effective at the recommended phase II dose (RP2D). In a higher dose almost 50% of patients exhibited severe (grade 3–4) neutropenia. A decrease in Bcl-2 protein levels in PBMCs was also observed. In conclusion, these results suggest that the addition of oblimersen may increase the chemotherapeutic cytotoxicity of irinotecan, but further testing with randomized trials is needed to evaluate the real efficacy of this combination treatment [16].

It is suggested that chemoresistance in metastatic melanoma can be attributed to high antiapoptotic activity by the Bcl-2 protein family. Under these circumstances, a randomized phase III trial was conducted in 2000 to compare the effectiveness of dacarbazine with or without oblimersen in advanced malignant melanoma. The overall survival (OS) time was not improved in the arm who received combined therapy, but the progression-free survival (PFS) time was significantly increased [17, 18]. No difference in treatment outcomes was observed in patients with elevated serum lactate dehydrogenase (LDH), suggesting that LDH can be used as a biomarker for adverse prognosis. Overall, this study indicates that oblimersen sodium can enhance the efficacy of dacarbazine in patients with advanced melanoma with normal baseline LDH levels [18].

Another randomized, phase II study in 2004 assessed the antitumor activity and safety of oblimersen sodium when administered before docetaxel versus docetaxel alone among patients with castration-resistant prostate cancer (CRPC). It has been found that Bcl-2 is upregulated in prostate cancer cells, leading to androgen independence and subsequent chemoresistance to docetaxel [19, 20]. Therefore, the addition of a Bcl-2 inhibitor could possibly enhance the sensitivity of CRPC cells to docetaxel. The results though failed to show a prostate-specific antigen (PSA) response greater than 30% or a major toxic event rate less than 45% in the oblimersen-docetaxel group. The combination treatment was also associated with a higher incidence of fatigue, mucositis, and thrombocytopenia. This study highlights the possibility of improved outcomes if the target group was more specific, as observed in the study above which proved that the population with normal LDH levels exhibited better response [21].

Although some studies exhibited a synergistic effect between oblimersen and specific chemotherapeutic agents, the results were not significant enough to continue examining this drug. The fact that the last study of oblimersen sodium in solid tumors we managed to find was more than a decade ago shows that this Bcl-2 inhibitor is probably no longer used in clinical trials.

### Obatoclax mesylate (GX15-070)

Unlike other more selective Bcl-2 inhibitors, obatoclax is considered a pan-Bcl-2 inhibitor, meaning that it targets all anti-apoptotic Bcl-2 family proteins [22]. Thanks to its ability to inhibit MCL-1, obatoclax was expected to have a promising antitumor effect on solid malignancies, especially in small-cell lung cancer (SCLC) where overexpression of MCL-1 is considered to cause insensitivity to other more selective Bcl-2 inhibitors [23]. In our study, we assessed five completed clinical trials which had used obatoclax mesylate in the presence of other agents for the treatment of solid cancers (Table 1).

In 2006, an open-label, phase I/II study was conducted in patients with NSCLC that relapsed after first-line platinum-based treatment. NSCLC cells are known to overexpress Bcl-2 antiapoptotic proteins [24], so the addition of a Bcl-2 inhibitor was expected to improve tumor sensitivity to taxanes. Obatoclax mesylate was combined with standard second-line NSCLC drug, docetaxel, to evaluate the tolerability and tumor response. Among the most common toxicities were neutropenia, as frequently observed with docetaxel, and transient neurologic side-effects, which is consistent with the findings of other clinical trials of obatoclax. No significant response rate was observed and thus, this study does not support further evaluation of the combination treatment in such patients [25].

In 2007, an open-label, single-arm phase II study evaluated the efficacy of combining topotecan with obatoclax mesylate in patients with relapsed SCLC. Topoisomerase inhibitors, like topotecan, trigger apoptosis by causing DNA damage to cancer cells. Antiapoptotic agents like the Bcl-2 family proteins can hinder this mechanism and lead to treatment resistance. Considering the above, a Bcl-2 inhibitor was expected to increase the chemotherapeutic effect. However, the addition of obatoclax did not manage to exceed the response rate of topotecan monotherapy in these patients. The most common adverse effects were neurologic, including ataxia and somnolence, lasting no more than 2 h. Hematologic toxicities were relatively infrequent, but more severe and required blood and platelet transfusion. Even so, it is worth mentioning that topotecan alone has a response rate as low as 7–10% in patients with platinum-refractory SCLC and, considering the small number of patients enrolled, it was not expected to observe a significantly higher efficacy [26].

In a more extended, randomized, phase II study in 2008 obatoclax mesylate was added to carboplatin/etoposide chemotherapy as first-line treatment in patients with extensive-stage SCLC. As expected, transient neurologic and psychiatric adverse effects were present in the obatoclax + carboplatin/etoposide arm, but no treatment discontinuation was needed. Unfortunately, the addition of obatoclax did not prove to significantly increase objective response rate [27].

In conclusion, obatoclax mesylate was not reported to add to the efficacy of other cytotoxic agents, neither in previously untreated nor in platinum-resistant lung cancer. To our knowledge, there are currently no recruiting clinical trials of obatoclax mesylate in patients with solid tumors.

### Navitoclax (ABT-263)

Navitoclax is one of the Bcl-2 family protein inhibitors which shows a high affinity to Bcl-2, Bcl-W and Bcl-xL antiapoptotic proteins. Early preclinical studies indicate that navitoclax alone is potent to cause suppression in tumors that rely on the overexpression of antiapoptotic Bcl-2 proteins for their survival, such as SCLC, acute lymphocytic leukemia (ALL) and fibrotic diseases [28]. In this review we examined a total of 12 completed clinical trials of navitoclax alone or with the presence of other drugs in solid tumor treatment (Table 1).

In 2007, a non-randomized Phase I study was conducted to evaluate tolerability, pharmacokinetics, and efficacy of navitoclax in SCLC and other solid tumors. Overall, navitoclax was well tolerated with most of treatment-related toxicities being grade 1–2. All patients experienced thrombocytopenia, a known adverse event of drugs that inhibit Bcl-xL, which was dose-dependent and manageable. This study also indicates that in some cases of SCLC, MCL-1 rather than Bcl-2/Bcl-xL is responsible for evading apoptosis. This might explain why these tumors show insensitivity to navitoclax and other Βcl-2/Bcl-xL inhibitors. Combination of navitoclax and other drugs that downregulate the expression of MCL-1 could subsequently have promising results in these tumors [23]. Phase II included the evaluation of safety at the RP2D, as well as the preliminary efficacy of navitoclax in patients with recurrent SCLC. The results were disappointing, as limited antitumor activity was achieved and thus, the majority of the patients discontinued treatment due to disease progression. Once again, the study recommends that future studies should focus on combination therapy [29].

In a prospective, multicenter Phase II study in 2015 navitoclax was tested as a single agent on women with heavily pretreated, platinum-resistant ovarian cancer (MONAVI-GINECO study), since a promising antitumor effect on chemo-resistant ovarian cancer cells was demonstrated during preclinical trials. Thrombocytopenia was the main side-effect, but it was reversible with no significant bleeding or toxicity-related deaths. Unfortunately, navitoclax in monotherapy exhibited no significant antitumor activity. No correlation between the expression of the pro-apoptotic BIM and the anti-apoptotic MCL-1 with disease progression was observed from the analysis of tumor biopsies [30].

In the same year, another Phase Ib study evaluated the safety and feasibility of navitoclax in combination with osimertinib among patients with epidermal growth factor receptor (EGFR)-mutant NSCLC who had exhibited resistance to prior tyrosine kinase inhibitor (TKI) exposure. Preclinical studies had shown that increased apoptotic activity significantly enhances the anti-tumor effect of a third generation TKI, possibly resulting in stronger and more long-lasting tumor regression in clinical models. Early thrombocytopenia was, as expected, the major adverse effect. However, this time the combination treatment not only proved to be safe, but also demonstrated clinical efficacy. It is indicated that further investigation of Bcl-2 inhibition and osimertinib combination is needed to validate these outcomes [31].

A low platelet count was reported as the principal side-effect in almost all clinical trials of navitoclax in monotherapy or in combination treatment. This is a result of navitoclax directly inducing the apoptotic death of platelets, as it inhibits their main anti-apoptotic factor, Bcl-xL [32]. Although in such cases thrombocytopenia occurrence was transient and well-tolerated, new and improved Bcl-2 family protein inhibitors needed to be developed to limit this toxicity.

### Palcitoclax (APG-1252)

Palcitoclax is a highly potent Bcl-2 family protein antagonist and, like navitoclax, targets mainly the Bcl-2 and Bcl-xL antiapoptotic proteins. It was developed in an effort to reduce on-target platelet toxicity while maintaining high anticancer activity. Preclinical studies have shown that palcitoclax can achieve tumor suppression in multiple xenograft models, including ALL, SCLC, colorectal and breast cancer [33, 34]. We examined 1 clinical trial of palcitoclax in solid tumors which was completed with available results (Table 1).

In 2017 the first in-human phase I study of palcitoclax was conducted to evaluate the safety, pharmacokinetics, and efficacy of the drug among US patients with metastatic SCLC or other solid malignancies [34]. Although palcitoclax was safe at doses lower than dose-limiting toxicity (DLT), with a relatively tolerable platelet toxicity, the supporting evidence is limited and further investigation is necessary to establish their possible antitumor effect.

### AT-101 (R-(-)-gossypol acetic acid)

Gossypol, a complex compound, naturally produced by cotton plants, was first discovered in the 1950s in China, as cooking with crude cottonseed was found to cause infertility in men [35, 36]. The gossypol (-)-enantiomer, known as gossypol acetic acid or AT-101, is the more biologically active form and it effectively induces the mitochondrial apoptotic pathway by downregulating the anti-apoptotic Bcl-2 proteins, including Bcl-2, Bcl-xL, Mcl-1, and Bcl-w [37, 38]. Except for the Bcl-2 inhibition pathway, AT-101 seems to play a significant role in the regulation of other cell signaling pathways by inhibiting vascular endothelial growth factor (VEGF)-mediated angiogenesis and Apurinic/apyrimidinic endodeoxyribonuclease 1 (APE1) [39, 40]. In our study, we summarized the outcomes of 14 completed clinical trials in which AT-101 was used to treat patients with solid cancers [Table 1].

In 2007 a double- blind, multicenter, randomized phase II study was conducted to evaluate the efficacy and tolerability of AT-101 in combination with docetaxel for the treatment of relapsed NSCLC. In preclinical prostate and lung cancer models, docetaxel and AT-101 were found to have a synergistic or even additive effect. However, in this clinical trial the combination treatment did not achieve an improved PFS. The adverse effects in the docetaxel + AT-101 arm were generally the same as in the docetaxel + placebo arm, with the exception of headache, which was significantly more frequent in the docetaxel + AT-101 group (11.3% compared to 0%). An increase in median OS by 1.9 months was reported in the AT-101 group, but it did not meet statistical significance [41].

Another randomized, double-blind, placebo-controlled, phase II study also in 2007 compared the potency of AT-101 in combination with docetaxel and prednisone (ADP arm) versus docetaxel and prednisone plus placebo (placebo-DP arm) in patients with chemotherapy-naïve metastatic hormone refractory prostate cancer. The ADP arm was associated with an increased incidence in cardiac and hematologic adverse events, as well as pulmonary embolism and peripheral neuropathy. Efficacy endpoints (especially OS and ≥ 50% PSA decline) were found to be increased in the subgroup of high-risk patients of the ADP arm, suggesting that Bcl-2 family protein expression may play a more significant role in such patients. However, no statistically significant difference in OS or PFS was observed between the two arms [42].

In 2012 a new therapeutic approach to advanced laryngeal cancer was tested with the combination of platinum, docetaxel and AT-101 in a randomized, phase II study. While adverse effects were overall more manageable, AT-101 did not improve disease response [43].

In a recent study with both preclinical and clinical aspects among patients with gastroesophageal cancer the combination of AT-101 with docetaxel, fluorouracil, and radiation exhibited surprisingly encouraging results, suggesting that the proapoptotic effect of AT-101 successfully overcomes the antiapoptotic pathways of gastroesophageal cancer stem cells [44]. However, more randomized, placebo-controlled clinical trials are needed to further investigate and confirm these results.

In conclusion, AT-101 was well-tolerated in all combination treatments, but in most studies improvement in primary endpoints did not reach statistical significance.

### Venetoclax (ABT-199)

In an effort to limit the adverse hematological toxicities of navitoclax, a new highly selective Bcl-2 inhibitor was introduced. Unlike other Bcl-2 inhibitors, which target more than one anti-apoptotic proteins, venetoclax is a selective inhibitor of the Bcl-2 protein and was first FDA approved in 2016 for the treatment of CLL, especially with 17p deletion [45]. It is now considered a novel drug for not only CLL, but also acute myeloid leukemia (AML) and small lymphocytic lymphoma, and it has exhibited promising results in other blood cancers, such as multiple myeloma [46]. However, the therapeutic potential of venetoclax in solid tumors is still under clinical evaluation. We assessed 2 completed clinical trials examining the use of venetoclax in different types of solid cancers with the one having available results (Table 1).

In 2018 a randomized, phase II study (VERONICA) compared the efficacy of venetoclax in combination with fulvestrant compared with fulvestrant alone in women with estrogen receptor (ER)-positive, HER2-negative, locally advanced or Metastatic Breast Cancer (MBC) who experienced disease recurrence or progression during or after treatment with CDK4/6i therapy. Preclinical studies support that Bcl-2 is overexpressed in approximately 85% of primary ER-positive breast cancer cases, so the inhibition of the antiapoptotic protein could enhance the therapeutic effect of endocrine therapy with fulvestrant in such patients. However, the clinical benefit rate and the PFS were not significantly improved in the venetoclax + fulvestrant arm. More serious (grade 3–4) adverse events were also observed in the combination treatment [47]. As of October 2020, participants in the venetoclax + fulvestrant arm have all discontinued venetoclax treatment and have continued on fulvestrant treatment alone [48].

### LP-118

LP-118 is an oral selective Bcl-2/Bcl-xL inhibitor and it is the newest of all drugs we have included in this study. Its special feature is that its anti Bcl-xL activity is adjusted to the minimum so that the risk of thrombocytopenia is limited [49]. We report only one recruiting clinical trial of LP-118 as a single agent in patients with lymphoma or solid tumors and the results are expected in the forthcoming years (Table 2).

**Table 2:** Active and recruiting clinical trials using Bcl-2 inhibitors in solid tumor treatment

Drug	Target	NCT	Trial design	Solid tumor description	Primary outcome
APG-1252 (Palcitoclax)	Bcl-2, Bcl-xL, Bcl-w	04893759	Open label, single group assignment phase I study	Neuroendocrine tumors	MTD Safety profile
ABT-263 (Navitoclax)	Bcl-2, Bcl-xL and Bcl-w	02079740	Open label, phase II STUDY	Metastatic Malignant Solid Neoplasm, Refractory Malignant Solid Neoplasm, Unresectable Malignant Solid Neoplasm	AE RR PFS
ABT-263 (Navitoclax)	Bcl-2, Bcl-xL and Bcl-w	02143401	Open label, phase I study	Metastatic Malignant Solid Neoplasm, Recurrent HCC, Recurrent Malignant Solid Neoplasm, Refractory Malignant Neoplasm, Stage IV HCC AJCC v7, Unresectable Solid Neoplasm	MTD AE
ABT-263 (Navitoclax)	Bcl-2, Bcl-xL and Bcl-w	03366103	Open label phase I/IIa study	SCLC and other solid tumors	AE ORR
ABT-263 (Navitoclax)	Bcl-2, Bcl-xL and Bcl-w	01989585	Randomized, parallel assignment, open label phase I/II study	BRAF mutant melanoma and other solid tumors	RP2D CR Maximal degree of tumor regression
ABT-199 (Venetoclax)	Bcl-2	03900884	Open-label, phase Ib study	Breast neoplasm female	MTD DLT
ABT-199 (Venetoclax)	Bcl-2	03751436	Open-label phase Ib/II	Metastatic castrate resistant prostate cancer	MTD RP2D PFS
ABT-199 (Venetoclax)	Bcl-2	03236857	Open-label, phase I	Neuroblastoma and other relapsed or refractory malignancies	AE DLT RP2D Cmax, Tmax
ABT-199 (Venetoclax)	Bcl-2	04553692	Open-label, phase I	Relapsed and/or refractory solid cancers	AE RP2D
LP-118 tablet	BCL-2/BCL-XL	05025358	Open-label, phase I	Solid tumor, Lymphoma, B-Cell	MTD AE RP2D Cmax, Tmax

*MTD* maximum tolerated dose, *AE* adverse event, *RR* response rate, *ORR* overall response rate, *PFS* progression-free survival, *HCC* hepatocellular carcinoma, *AJCC* American joint committee on cancer, *SCLC* small-cell lung cancer, *RP2D* recommended phase 2 dose, *CR* complete response, *DLT* dose-limiting toxicity

## Interpretation and discussion

As noted earlier, Bcl-2 inhibitors have proven their effectiveness in treating hematological malignancies through multiple preclinical and clinical studies for many years now. Their crucial role in evading apoptosis still renders them as one of the most promising targets for cancer treatment. Early data from preclinical studies have supported that some Bcl-2 family proteins are overexpressed in many solid tumors too, raising a big question; are Bcl-2 inhibitors as effective in solid tumors as in hematological? To answer this question, we examined a great number of clinical trials using the Bcl-2 inhibitors oblimersen sodium, obatoclax mesylate, navitoclax, palcitoclax, AT-101, venetoclax and LP-118. The most frequently utilized approach seems to be the co-administration of a Bcl-2 inhibitor with other anticancer agents, such as but not limited to conventional chemotherapy.

Oblimersen sodium and obatoclax mesylate did not manage to demonstrate statistically significant clinical efficacy in any case of solid malignancies. Further investigation is not suggested, and we report no recruiting clinical trials of these agents. AT-101 was found to successfully target cancer stem cells of gastroesophageal cancer in one clinical trial [44] but failed to prove significant antitumor effect in all other trials. Navitoclax was mainly tested in lung cancer and was expected to sufficiently increase apoptotic activity and chemosensitivity, as demonstrated in preclinical studies. Unfortunately, so far only one study has managed to prove statistically significant antitumor response [31]. Regarding palcitoclax, we cannot reach any conclusions based on one completed trial. Nevertheless, the first results as a monotherapy in metastatic SCLC were promising and further investigation is warranted [34]. Venetoclax is a newly developed drug and, therefore, little research is still conducted concerning solid malignancies. Many clinical trials are currently evaluating the efficacy of adding venetoclax to other agents in many types of solid tumors, such as CRPC, breast cancer and neuroblastoma.

In terms of overall safety and toxicity, the adverse events that occurred could be explained by both the co-administered agent and the Bcl-2 inhibitor itself. More specifically, common toxicities included severe neutropenia and thrombocytopenia (grade 3 and 4), anemia, lymphopenia, fatigue, diarrhea, vomiting, hepatic and metabolic disturbances. However, few instances of serious adverse events such as myocardial infarction and pulmonary embolism were recorded. Neurologic adverse events were reported only in groups receiving palcitoclax, but were transient and did not require any intervention. Thrombocytopenia was more prominent with navitoclax and was attributed to direct inhibition of Bcl-xL. Results from most randomized trials exhibited a higher incidence of hematologic toxicities among patients receiving combination treatment compared to conventional chemotherapy. Although these adverse events were manageable, it is suggested that the addition of Bcl-2 inhibitors to chemotherapeutic agents can aggravate the hematologic toxicities.

The main hindrance in evaluating the efficacy of these drugs was the limited number of participants, which predisposes to statistical errors, while a non-negligible number of patients had other comorbidities that could potentially aggravate the rate and severity of occurring adverse events. It is also worth mentioning that most trials were completed 10 or more years ago. Since then, patient care has evolved substantially and a variety of new chemotherapeutic agents has been released. These observations emphasize the necessity for renewed and more extended randomized clinical trials with stricter eligibility criteria regarding patients’ health status, as a means of objectively identifying clinical efficacy and safety issues.

## Conclusion

To our knowledge, this is the most recent review to summarize clinical trials of Bcl-2 inhibitors on solid tumors. It is evident that Bcl-2 inhibitors do not seem to be as efficient against solid tumors as they are against hematological cancers, when used as single agents. However, in combination with other anticancer drugs they are likely to enhance their antitumor effect while maintaining a good safety profile. We also highlight the importance of further preclinical research which may pave the way for new, more potent combinations of Bcl-2 inhibitors with other targeted agents. Currently 10 clinical trials are recruiting with their primary endpoints being the assessment of maximum tolerated dose, safety and adverse events, pharmacokinetic, pharmacodynamic parameters and effectiveness (Table 2). Their results are anticipated in the forthcoming years and should add useful information in our arsenal against cancer.

## Data Availability

Data are included in the manuscript.

## References

[1] Vogelstein B, Kinzler KW (2004). Cancer genes and the pathways they control. Nat Med.

[2] Sarkar S, Horn G, Moulton K, Oza A, Byler S, Kokolus S (2013). Cancer development, progression, and therapy: an epigenetic overview. Int J Mol Sci.

[3] Meier P, Finch A, Evan G (2000). Apoptosis in development. Nature.

[4] Hengartner MO (2000). The biochemistry of apoptosis. Nature.

[5] Montero J, Letai A (2018). Why do BCL-2 inhibitors work and where should we use them in the clinic?. Cell Death Differ.

[6] Ryan CE, Davids MS (2019). BCL-2 inhibitors, present and future. Cancer J.

[7] Hockenbery D, Nuñez G, Milliman C, Schreiber RD, Korsmeyer SJ (1990). Bcl-2 is an inner mitochondrial membrane protein that blocks programmed cell death. Nature.

[8] Pentimalli F (2018). BCL2: a 30-year tale of life, death and much more to come. Cell Death Differ.

[9] Hafezi S, Rahmani M (2021). Targeting BCL-2 in cancer: advances, challenges, and perspectives. Cancers (Basel).

[10] Anderson MA, Deng J, Seymour JF, Tam C, Kim SY, Fein J (2016). The BCL2 selective inhibitor venetoclax induces rapid onset apoptosis of CLL cells in patients via a TP53-independent mechanism. Blood.

[11] Frankel SR (2003). Oblimersen sodium (G3139 Bcl-2 antisense oligonucleotide) therapy in Waldenstrom’s macroglobulinemia: a targeted approach to enhance apoptosis. Semin Oncol.

[12] Klasa RJ, Gillum AM, Klem RE, Frankel SR (2002). Oblimersen Bcl-2 antisense: facilitating apoptosis in anticancer treatment. Antisense Nucleic Acid Drug Dev.

[13] Nakamura T, Nomura S, Sakai T, Nariya S (1997). Expression of bcl-2 oncoprotein in gastrointestinal and uterine carcinomas and their premalignant lesions. Hum Pathol.

[14] Valassiadou KE, Stefanaki K, Tzardi M, Datseris G, Georgoulias V, Melissas J (1997). Immunohistochemical expression of p53, bcl-2, mdm2 and waf1/p21 proteins in colorectal adenocarcinomas. Anticancer Res.

[15] Mueller J, Mueller E, Hoepner I, Jütting J, Bethke B, Stolte M (1996). Expression of bcl-2 and p53 in de novo and ex-adenoma colon carcinoma: a comparative immunohistochemical study. J Pathol.

[16] Mita MM, Ochoa L, Rowinsky EK, Kuhn J, Schwartz G, Hammond LA (2006). A phase I, pharmacokinetic and biologic correlative study of oblimersen sodium (Genasense™, G3139) and irinotecan in patients with metastatic colorectal cancer. Ann Oncol.

[17] Oblimersen. Drugs in R & D. 2007;8:321–34. 10.2165/00126839-200708050-00006 10.2165/00126839-200708050-00006 17767397

[18] Bedikian AY, Millward M, Pehamberger H, Conry R, Gore M, Trefzer U (2006). Bcl-2 antisense (oblimersen sodium) plus dacarbazine in patients with advanced melanoma: the oblimersen melanoma study group. J Clin Oncol.

[19] Gleave ME, Miayake H, Goldie J, Nelson C, Tolcher A (1999). Targeting bcl-2 gene to delay androgen-independent progression and enhance chemosensitivity in prostate cancer using antisense bcl-2 oligodeoxynucleotides. Urology.

[20] Goodin S, Rao Kv, DiPaola RS (2002). State-of-the-art treatment of metastatic hormone-refractory prostate cancer. Oncologist.

[21] Sternberg CN, Dumez H, van Poppel H, Skoneczna I, Sella A, Daugaard G (2009). Docetaxel plus oblimersen sodium (Bcl-2 antisense oligonucleotide): an EORTC multicenter, randomized phase II study in patients with castration-resistant prostate cancer. Ann Oncol.

[22] Vogler M (2014). Targeting BCL2-proteins for the treatment of solid tumours. Adv Med.

[23] Gandhi L, Camidge DR, de Oliveira MR, Bonomi P, Gandara D, Khaira D (2011). Phase I Study of navitoclax (ABT-263), a novel Bcl-2 family inhibitor, in patients with small-cell lung cancer and other solid tumors. J Clin Oncol.

[24] Berrieman HK, Smith L, O’Kane SL, Campbell A, Lind MJ, Cawkwell L (2005). The expression of Bcl-2 family proteins differs between nonsmall cell lung carcinoma subtypes. Cancer.

[25] Chiappori A, Williams C, Northfelt DW, Adams JW, Malik S, Edelman MJ (2014). Obatoclax mesylate, a Pan–Bcl-2 inhibitor, in combination with docetaxel in a phase 1/2 trial in relapsed non–small-cell lung cancer. J Thorac Oncol.

[26] Paik PK, Rudin CM, Pietanza MC, Brown A, Rizvi NA, Takebe N (2011). A phase II study of obatoclax mesylate, a Bcl-2 antagonist, plus topotecan in relapsed small cell lung cancer. Lung Cancer.

[27] Langer CJ, Albert I, Ross HJ, Kovacs P, Blakely LJ, Pajkos G (2014). Randomized phase II study of carboplatin and etoposide with or without obatoclax mesylate in extensive-stage small cell lung cancer. Lung Cancer.

[28] Mohamad Anuar NN, Nor Hisam NS, Liew SL, Ugusman A (2020). Clinical review: navitoclax as a pro-apoptotic and anti-fibrotic agent. Front Pharmacol.

[29] Rudin CM, Hann CL, Garon EB, de Oliveira MR, Bonomi PD, Camidge DR (2012). Phase II study of single-agent navitoclax (ABT-263) and biomarker correlates in patients with relapsed small cell lung cancer. Clin Cancer Res.

[30] Joly F, Fabbro M, Follana P, Lequesne J, Medioni J, Lesoin A (2022). A phase II study of navitoclax (ABT-263) as single agent in women heavily pretreated for recurrent epithelial ovarian cancer: the MONAVI—GINECO study. Gynecol Oncol.

[31] Bertino EM, Gentzler RD, Clifford S, Kolesar J, Muzikansky A, Haura EB (2021). Phase IB study of osimertinib in combination with navitoclax in *EGFR* -mutant NSCLC following resistance to initial *EGFR* therapy (ETCTN 9903). Clin Cancer Res.

[32] Debrincat MA, Pleines I, Lebois M, Lane RM, Holmes ML, Corbin J (2015). BCL-2 is dispensable for thrombopoiesis and platelet survival. Cell Death Dis.

[33] Yao W, Bai L, Wang S, Zhai Y, Sun S-Y (2022). Mcl-1 levels critically impact the sensitivities of human colorectal cancer cells to APG-1252-M1, a novel Bcl-2/Bcl-XL dual inhibitor that induces Bax-dependent apoptosis. Neoplasia.

[34] Lakhani NJ, Rasco DW, Zeng Q, Tang Y, Liang Z, Wang H (2020). First-in-human study of palcitoclax (APG-1252), a novel dual Bcl-2/Bcl-xL inhibitor, demonstrated advantages in platelet safety while maintaining anticancer effect in US patients with metastatic solid tumors. J Clin Oncol.

[35] Renner O, Mayer M, Leischner C, Burkard M, Berger A, Lauer UM (2022). Systematic review of gossypol/AT-101 in cancer clinical trials. Pharmaceuticals.

[36] Hoshiai H, Uehara S, Mori R, Nagaike F, Tsuiki A, Suzuki M (1982). Gossypol as oral contraceptive for male: trial case report. Tohoku J Exp Med.

[37] Cao H, Sethumadhavan K, Cao F, Wang TTY (2021). Gossypol decreased cell viability and down-regulated the expression of a number of genes in human colon cancer cells. Sci Rep.

[38] Zeng Y, Ma J, Xu L, Wu D (2019). Natural product gossypol and its derivatives in precision cancer medicine. Curr Med Chem.

[39] Pang X, Wu Y, Wu Y, Lu B, Chen J, Wang J (2011). (−)-Gossypol suppresses the growth of human prostate cancer xenografts via modulating VEGF signaling-mediated angiogenesis. Mol Cancer Ther.

[40] Wang D, Li M, Sui J, Ren T, Li Z, Zhang L (2014). Identification of a novel potential antitumor activity of gossypol as an APE1/Ref-1 inhibitor. Drug Des Devel Ther.

[41] Ready N, Karaseva NA, Orlov Sv, Luft Av, Popovych O, Holmlund JT (2011). Double-blind, placebo-controlled, randomized phase 2 study of the proapoptotic agent AT-101 plus docetaxel, in second-line non-small cell lung cancer. J Thorac Oncol.

[42] Sonpavde G, Matveev V, Burke JM, Caton JR, Fleming MT, Hutson TE (2012). Randomized phase II trial of docetaxel plus prednisone in combination with placebo or AT-101, an oral small molecule Bcl-2 family antagonist, as first-line therapy for metastatic castration-resistant prostate cancer. Ann Oncol.

[43] Swiecicki P, Bellile E, Casper K, Malloy KM, Kupfer R, Spector ME (2019). A randomized trial of laryngeal organ preservation evaluating two cycles of induction chemotherapy with platinum, docetaxel, and a novel Bcl-xL inhibitor. J Clin Oncol.

[44] Song S, Chen Q, Li Y, Lei G, Scott A, Huo L (2021). Targeting cancer stem cells with a pan-BCL-2 inhibitor in preclinical and clinical settings in patients with gastroesophageal carcinoma. Gut.

[45] Deeks ED (2016). Venetoclax: first global approval. Drugs.

[46] Juárez-Salcedo LM, Desai V, Dalia S (2019). Venetoclax: evidence to date and clinical potential. Drugs Context.

[47] Lindeman GJ, Bowen R, Jerzak KJ, Song X, Decker T, Boyle FM (2021). Results from VERONICA: a randomized, phase II study of second-/third-line venetoclax (VEN) + fulvestrant (F) versus F alone in estrogen receptor (ER)-positive, HER2-negative, locally advanced, or metastatic breast cancer (LA/MBC). J Clin Oncol.

[48] Lindeman GJ, Fernando TM, Bowen R, Jerzak KJ, Song X, Decker T (2022). VERONICA: randomized phase II study of fulvestrant and venetoclax in ER-Positive metastatic breast cancer post-CDK4/6 inhibitors—efficacy, safety, and biomarker results. Clin Cancer Res.

[49] Ravikrishnan J, Muhowski EM, Lai T-H, Misra S, Diaz Rohena D, Tan F (2021). Characterization of LP-118, a novel small molecule inhibitor of Bcl-2 and Bcl-Xl in chronic lymphocytic leukemia resistant to venetoclax. Blood.

